# An Assessment of Patient Satisfaction With Home Care Services in an Eastern City of Turkey

**DOI:** 10.1002/nop2.70214

**Published:** 2025-05-07

**Authors:** Ezgi Yarasir, Irem Bulut

**Affiliations:** ^1^ Department of Therapy and Rehabilitation Vocational School of Health Services, Firat University Elazig Turkey; ^2^ Department of Public Health Firat University Faculty of Medicine Elazig Turkey

**Keywords:** health status, home care services, nursing, patient satisfaction, patients

## Abstract

**Aim:**

The aim of this study was to examine the socioeconomic characteristics of patients who are receiving home care in an eastern city of Turkey, assess how satisfied they are with these services and determines the factors affecting their level of satisfaction.

**Design:**

A quantitative descriptive and cross‐sectional study design was used.

**Methods:**

From July to August 2022, a sample of 306 patients who were receivers of home care services participated in the research. The study comprised of a ‘Personal Information Form’ and the ‘Home Health Care Services Patient Experience Questionnaire’ supplied by the Turkish Ministry of Health, Department of Quality, Accreditation, and Employee Rights. The questionnaire evaluates the degree of satisfaction with home care services. The statistical analysis was performed using the SPSS 22.0 software. A significance level of *p* < 0.05 was utilised to determine statistical significance.

**Results:**

Out of all the participants, 51.3% were female and the average age was 74.1 ± 14.4 years. An impressive 92.8% of participants expressed their satisfaction with the home care services provided by the Ministry of Health, considering it highly effective. The mean satisfaction score of the participants in home care was determined to be 4.7 ± 0.3. The study found that participants who had completed at least a high school education, were part of large families, had a high monthly income and perceived their health as poor reported significantly greater levels of satisfaction with home care services (*p* < 0.05).

**Patient or Public Contribution:**

The term patient satisfaction remains of significant importance in the professions of nursing and health services alike. The results of our study indicated that nearly all of the participants expressed that they were satisfied with their use of home care. Using regular satisfaction surveys is advised to determine the underlying reasons for dissatisfaction among patients who are unsatisfied with the service, and to conduct research with the goal of implementing improvements.

## 1 Introduction

Home care services refer to the providing of healthcare services in the comfort of the patient's own residence with the aim of improving and maintaining individuals' health condition (Goger et al. 2023). It is a practice that aims to enhance patients' living conditions, decrease the excessive use of hospital services and reduce the rising expenses of the healthcare system by delivering medical treatments to patients in their own homes (Mascolo et al. 2017). The demand for home care services is growing globally. All stakeholders, including patients, their relatives, health institutions, governments and insurance companies, play a role in the growing utilisation of home care services.

Home care services are commonly connected with geriatric services due to its primary focus on providing care to elderly people. The World Health Organization (WHO) estimates the global elderly (65+) population will reach 2 billion by 2050, with 80% of this demographic residing in developing nations (World Health Organization 2024). According to estimates from the Turkish Statistical Institute (TSI), the proportion of senior people in Turkey is projected to be 11.0% in 2025, 12.9% in 2030, 16.3% in 2040, 22.6% in 2060 and 25.6% in 2080, aligning with global trends (Turkish Statistical Institute 2023). Several studies conducted in Turkey indicate that the majority of individuals utilising home care services are patients who are 60 years or older. Some key contributions to why the demand for home care has increased are the rising expenses associated with healthcare services provided in hospitals, the ongoing requirement for care following discharge and the emergence of various additional healthcare needs due to the aging population have contributed to the increased demand for home care services. Consequently, the preference for home care has led to a reduction in hospital visits and the duration of hospital stays (Diseases TIoPHaC 2022). Providing home care services to individuals in their own residence or living environment has several advantages. It helps to minimise healthcare costs, ensures that the individual receives personalised care, fosters family unity in the caregiving process, and educates patients and their families about healthcare. Therefore, this approach offers significant benefits for both the individual and society as a whole (Altuntas et al. 2010). Research indicates that home care services have a positive impact on patient outcomes and lead to cost savings (Federman et al. 2023). The provision of home care services commenced in Turkey in 1993 and experienced significant growth after 2004 (Altuntas et al. 2010). The provision of these services is mostly carried out by local governments, private hospitals, private home care facilities and public hospitals (Goger et al. 2023).

The term of patient satisfaction was created for the benefit of the individuals who derive advantages from healthcare services. Put simply, patients have some predetermined expectations regarding the service they receive. Whether individuals experience satisfaction or discontent is contingent upon whether the outcome they obtain aligns with their expectations (Meral 2006). Various uncontrolled factors, including patients' and their relatives' prior experiences with their diseases, the service delivery method of health institutions, sociocultural situations and perceptions, physical environment, trust and wages, can significantly impact the satisfaction process of patients and their relatives. Assessing patient satisfaction is a crucial factor in evaluating the excellence of healthcare services delivered in home care settings (Goger et al. 2023).

According to Oksholm et al. (2023), there is evidence suggesting that patient satisfaction is linked to higher adherence to doctor advice and follow‐up consultations, resulting in better health outcomes. Studies in the literature have reported that patient satisfaction with home care services ranges from 30.5% to 84.0% (Oksuz 2018; Guduk et al. 2020; Dawani et al. 2014). A randomised controlled trial conducted on homebound patients to evaluate the effect of a home health intervention demonstrated that satisfaction was 2.26 times greater in the group receiving the intervention, with a 95% confidence interval ranging from 1.46 to 3.06 (Federman et al. 2023).

Patient satisfaction is a crucial metric for assessing the quality of healthcare delivered to patients. Continuous and regular evaluation of patient satisfaction using accurate assessment instruments is crucial. Currently, there is a lack of universally recognised approaches or measuring methodologies for assessing patient satisfaction (Batbaatar et al. 2017). This study aims to assess the satisfaction levels of patients receiving home care services and analyse the factors that influence individual satisfaction.

## 2 Methods

### 2.1 Type of Study

This descriptive, and cross‐sectional study was conducted in Elazig, an eastern province of Turkey with a population of 604 thousand. The patients in this study get home care services from the Turkish Ministry of Health, which is a component of the National Health Service. These services are offered to the patients at no cost. The research's fieldwork was carried out over the months of July and August in 2022.

### 2.2 Participants

The study population consists of 5386 patients that are enrolled in the home care service in Elazig. The study sample size was determined to be 352 persons using the Epi Info program. The population size was 5386, and the case prevalence was estimated to be 57.2% (with a range of 30.5%–84.0%) based on previous studies (Oksuz 2018; Guduk et al. 2020; Dawani et al. 2014). The study aimed to achieve a 95% confidence interval with a 5% error rate. A total of 306 participants were reached during the study, representing 86.9% of the sample. The eligibility criteria for participation in the study are as follows: participants must be 18 years of age or older, willing to receive home care services and capable of actively engaging in the study. The exclusion criteria for the study includes lack of willingness to participate or a cognitive disability that hinders comprehension and response to survey questions.

### 2.3 Data Collection Tools

The research included a questionnaire form consisting of two separate parts for collecting data. The researchers filled the questionnaire form utilising the face‐to‐face survey method. The survey completed in approximately 10–15 min to complete.

#### 2.3.1 Personal Information Form

The personal information form was created by researchers and consists of 37 questions based on relevant literature sources (Goger et al. 2023; Gey and Yarar 2019). The questionnaire considered the age, gender, marital status, income level, education level, family type, chronic disease status, smoking and alcohol habits, and characteristics of home care services of the participants.

#### 2.3.2 Home Health Care Services Patient Experience Questionnaire

The ‘Home Health Services Patient Experience Survey’ developed by the Department of Health Quality, Accreditation, and Employee Rights of the Turkish Ministry of Health was utilised to assess the satisfaction of the participants with home care services (Home Health Care Patient Experience Survey 2022). The Likert scale survey comprises 14 questions, each rated on a five‐point scale. The response options range from “Strongly Disagree (1)” to “Strongly Agree (5)”. The scale does not include any items that are scored in reverse. A higher average score on the scale shows an increase in patient satisfaction.

### 2.4 Statistical Analysis

The statistical analysis of the study's findings was conducted using the SPSS 22.0 software package. According to the Kolmogorov–Smirnov test, non‐parametric tests were used due to the data distribution did not conform to normal distribution. The statistical evaluations used percentage, median, Mann–Whitney *U* test, Kruskall–Wallis test, Bonferroni Test and Spearman correlation analysis. The means are shown with their corresponding standard deviations (mean ± SD). A *p*‐value of less than 0.05 was regarded as indicating statistical significance.

## 3 Results

Out of the total number of participants, 51.3% (*n* = 157) were women. The average age of the participants was 74.1 ± 14.4 years, with the youngest participant being 25 years old and the oldest being 97 years old. A percentage of 41.5% illiterate literacy and reading skills, whereas 51.6% were in a state of married. Table 1 shows the distribution of patients receiving home care services based on their sociodemographic characteristics.

**TABLE 1:** Distribution of patients receiving home care services according to sociodemographic characteristics.

Sociodemographic characteristics (*n* = 306)	*n*	%
Gender		
Female	157	51.3
Male	149	48.7
Age		
25–64	48	15.7
65–74	85	27.8
75–84	96	31.4
85–97	77	25.1
Marital status		
Single	16	5.2
Married	158	51.6
Widow	125	40.8
Divorced	7	2.4
Educational status		
Illiterate	127	41.5
Literate	98	32.0
Primary school graduate	39	12.7
Secondary school graduate	20	6.5
High school graduate and above	22	7.3
Family type		
Nuclear	229	74.8
Extended	77	25.2
Presence of children at home		
Yes	86	28.1
No	220	71.9
Monthly income level^a^		
3000 Turkish Liras and below	60	19.6
3001–5500 Turkish Liras	205	67.0
5501 Turkish Liras and above	41	13.4
Social insurance		
No	50	16.3
General health insurance	249	81.4
Private health insurance	7	2.3
Smoking		
Yes	29	9.5
Quit	119	38.9
Never	158	51.6
Alcohol use		
Yes	5	1.6
No	301	98.4
Presence of additional chronic disease		
Yes	207	67.6
No	99	32.4
Perception of health status		
Good	9	2.9
Moderate	189	61.8
Poor	108	35.3

^a^ The minimum wage was 5500 TL on the dates of the field work of the research.

It was reported by participants that an average of 1.2 ± 0.6 days following the initial request for home care services was the mean elapsed time for the administration of home visits to assess their requests. Patients began receiving home care services within an average of 1.2 ± 0.7 days following the initial application. The participants had been getting home care for an average of 3.0 ± 1.9 years. A staggering 92.8% of respondents express their unequivocal satisfaction with the home care service offered by the Ministry of Health. Table 2 provides the breakdown of participants based on their health characteristics.

**TABLE 2:** Distribution of patients receiving from home health services according to their health characteristics.

Health characteristics (*n* = 306)	*n*	%
Diagnosis		
Cardiovascular system diseases	83	27.1
Respiratory system diseases	36	11.8
Musculoskeletal system diseases	69	22.5
Neurological system diseases	96	31.4
Cancer	22	7.2
Have you ever had COVID‐19?		
Yes	207	67.6
No	99	32.4
Eating regularly		
Yes	212	69.3
No	94	30.7
Number of daily meals		
1–2	67	21.9
3	212	69.3
4–6	27	8.8
Status of knowing the family physician		
Yes	176	57.5
No	130	42.5
The place to learn home care services		
Social environment	146	47.7
Family physician	73	23.9
Healthcare facility	68	22.2
Media	19	6.2
The place where you applied for home care		
By calling home care services	268	87.6
Visiting to a home care service center	27	8.8
By family physician	11	3.6
Medical staff who come for the first examination in home care services^a^		
Nurse	298	97.4
Doctor	243	79.4
Physiotherapist	4	1.3
Psychologist	2	0.7
How many days did it take for the home care service to come for the first examination visit after the initial application?		
1 day	248	81.0
2 days	50	16.3
3–7 days	8	2.7
Time from first examination to start of home care		
1 day	242	79.1
2 days	48	15.7
3–7 days	16	5.2
Does the home care team call you before they arrive?		
Yes	281	91.8
No	25	8.2
Is the environment where you are examined comfortable at home?		
Yes	302	98.7
No	4	1.3
Are you satisfied with the doctor who examined you?		
Yes	301	98.4
No	5	1.6
Perception of home care service provided by the Ministry of Health		
The service provided is successful and satisfactory.	284	92.8
The service provided is incomplete but satisfactory.	8	2.6
The service provided met my expectations.	12	3.9
The service provided did not meet my expectations.	2	0.7

^a^ More than one answer could be marked.

Several characteristics associated with the participants' own caregivers included the following: The mean age was 46.7 ± 12.2, with 76.5% of the participants being women. Additionally, 89.5% had a high school education or below, 60.5% were first degree relatives of the patient and 73.5% did not have any employment other than patient care. The distribution of sociodemographic characteristics of caregivers is shown in Table 3.

**TABLE 3:** Distribution of sociodemographic characteristics of caregivers.

The caregiver's sociodemographic characteristics (*n* = 306)	*n*	%
Gender		
Female	234	76.5
Male	72	23.5
Age		
26–35	37	12.1
36–45	131	42.8
46–54	79	25.8
55–85	59	19.3
The caregiver's closeness to the patient		
First degree relative	185	60.5
Second degree relative	62	20.2
Spouse	53	17.3
Paid caregiver	6	2.0
Educational status		
Illiterate	34	11.1
Literate	98	32.0
Primary school degree	36	11.8
Secondary school degree	30	9.8
High school degree	76	24.8
University degree	32	10.5
Working situation other than caregiving		
No	225	73.5
Full time	59	19.3
Part time	22	7.2

The mean score of the participants on the Home Health Services Patient Experience Survey scale was determined to be 4.7 ± 0.3. Table 4 displays the distribution of participants' responses to the survey measuring the patient experience in home care.

**TABLE 4:** Distribution of participants' answers to the home health care services patient experience questionnaire.

Home healthcare patient experience questionnaire	Totally agree		Agree		Undecided		Disagree		Totally disagree	
	*n*	%	*n*	%	*n*	%	*n*	%	*n*	%
I had no difficulties in the application process for health care services	241	78.8	61	19.9	2	0.7	2	0.7	—	—
After the application, the first review was completed within a week	229	74.8	63	20.6	2	0.7	—	—	12	3.9
During the first visit, sufficient information was given about the service to be provided	233	76.1	73	23.9	—	—	—	—	—	—
Adequate information is given about the visit dates	221	72.2	83	27.1	2	0.7	—	—	—	—
I receive service on specified visit dates	215	70.3	89	29.1	2	0.7	—	—	—	—
I can easily access the unit whenever I need it	215	70.3	87	28.4	4	1.3	—	—	—	—
My requests and problems are listened to carefully	224	73.2	80	26.1	2	0.7	—	—	—	—
My doctor allocates enough time for the examination	218	71.2	69	22.5	19	6.2	—	—	—	—
My doctor provides adequate information about my disease and treatment	237	77.5	69	22.5	—	—	—	—	—	—
My personal privacy is taken care of during examinations	249	81.4	57	18.6	—	—	—	—	—	—
I am informed about the laboratory analysis process, and results	225	73.5	79	25.8	2	0.7	—	—	—	—
The service personnel are kind to me	235	76.8	69	22.5	2	0.7	—	—	—	—
The services provided by the home health care unit met my expectations	218	71.2	88	28.8	—	—	—	—	—	—
I recommend home health care services to anyone who needs them	218	71.2	88	28.8	—	—	—	—	—	—

The satisfaction scores for participants receiving health care services did not show any significant change based on gender, age and marital status (*p* > 0.05, Table 5). The study found that participants who had completed at least a high school education, came from large families, had a high monthly income and perceived their health as poor reported considerably higher levels of satisfaction with home care services (*p* < 0.05). The satisfaction levels of participants only providing care for the patient at home were significantly greater compared to those employed in a different occupation (*p* < 0.05).

**TABLE 5:** Distribution of satisfaction scores according to sociodemographic characteristics of participants.

Sociodemographic characteristics (*n* = 306)	*n*	Home health care satisfaction score median (min–max)	Statistics^b^
Gender			
Female	157	4.92 (3.71–5.00)	*U* = 11654.500 *p* = 0.953
Male	149	5.00 (3.50–5.00)	
Age			
25–64	48	4.89 (4.29–5.00)	*x* ^2^ = 0.472 *p* = 0.925
65–74	85	5.00 (3.71–5.00)	
75–84	96	5.00 (3.50–5.00)	
85–97	77	4.85 (3.71–5.00)	
Marital status			
Single	16	4.71 (4.50–5.00)	*x* ^2^ = 1.684 *p* = 0.641
Married	158	5.00 (3.50–5.00)	
Widow	125	5.00 (3.71–5.00)	
Divorced	7	4.92 (4.93–5.00)	
Educational status			
Illiterate	127	4.92 (3.50–5.00)	*x* ^2^ = 18.878 ***p* = 0.001**
Literate	98	5.00 (4.00–5.00)^c^	
Primary school graduate	39	4.57 (3.71–5.00)^c^	
Secondary school graduate	20	4.92 (4.14–5.00)	
High school graduate and above	22	5.00 (3.86–5.00)^c^	
Family type			
Nuclear	229	4.85 (3.50–5.00)	*U* = 6060.000 ***p* < 0.001**
Extended	77	5.00 (4.43–5.00)	
Presence of children at home			
Yes	86	5.00 (3.71–5.00)	*U* = 7604.000 ***p* = 0.004**
No	220	4.85 (3.50–5.00)	
Monthly income level^a^			
3000 Turkish Liras and below	60	4.96 (3.93–5.00)	*x* ^2^ = 6.896 ***p* = 0.032**
3001–5500 Turkish Liras	205	5.00 (3.50–5.00)^c^	
5501 Turkish Liras and above	41	5.00 (4.50–5.00)^c^	
Social insurance			
No	50	5.00 (4.00–5.00)	*U* = 4931.000 ***p* = 0.006**
Yes (General/private health insurance)	256^d^	4.92 (3.50–5.00)	
Smoking			
Yes	29	5.00 (4.57–5.00)^c^	*x* ^2^ = 9.914 ***p* = 0.007**
Quit	119	4.85 (3.50–5.00)^c^	
Never	158	5.00 (3.71–5.00)	
Does your own caregiver work in another job besides caregiving?			
Yes (Full time‐part time)	225	4.92 (3.50–5.00)	*U* = 7686.500 ***p* = 0.024**
No	81	5.00 (4.14–5.00)	

^a^ The minimum wage was 5500 TL on the dates of the field work of the research.

^b^ The Mann–Whitney *U* test was used to compare two independent groups, and the Kruskal–Wallis test was used to compare more than two groups.

^c^ Groups where the difference originates (Bonferroni test).

^d^ *Note*: Bold values indicate statistical significance of *P* < 0.05.

Participants with neurological system disease and a self‐perceived poor health status had greater satisfaction levels (*p* < 0.05). Table 6 shows the distribution of satisfaction scores based on the health characteristics of the participants.

**TABLE 6:** Distribution of satisfaction scores according to participants' health characteristics.

Health characteristics (*n* = 306)	*n*	Home health care satisfaction score median (min–max)	Statistics^a^
Diagnosis			
Cardiovascular system diseases	83	4.71 (3.71–5.00)^b^	*x* ^2^ = 16.170 ***p* = 0.003**
Respiratory system diseases	36	4.85 (4.43–5.00)	
Musculoskeletal system diseases	69^c^	4.92 (3.71–5.00)	
Neurological system diseases	96	5.00 (3.86–5.00)^b^	
Cancer	22	4.92 (3.50–5.00)	
Presence of additional chronic disease			
Yes	207	5.00 (3.50–5.00)	*U* = 7556.500 ***p* < 0.001**
No	99	4.71 (3.71–5.00)	
Perception of health status			
Good	9	5.00 (4.14–5.00)	*x* ^2^ = 8.294 ***p* = 0.016**
Moderate	189	4.92 (3.71–5.00)^b^	
Poor	108	5.00 (3.50–5.00)^b^	
Status of knowing the family physician			
Yes	176	4.89 (3.71–5.00)	*U* = 9523.000 ***p* = 0.007**
No	130	5.00 (3.50–5.00)	
The place to learn home care			
Family physician	73	4.78 (3.71–5.00)^b^	*x* ^2^ = 35.665 ***p* < 0.001**
Social environment	146	5.00 (3.86–5.00)^b^	
Media	19	5.00 (4.71–5.00)	
Healthcare facility	68	4.71 (3.50–5.00)^b^	
The place where you applied for home care			
By calling home care services	268	5.00 (3.71–5.00)	*x* ^2^ = 2.975 *p* = 0.226
Going to a home care centre	27	4.78 (3.86–5.00)	
Contacting your family doctor	11	4.92 (3.50–5.00)	
How many days did it take for the first examination?			
1 day	248	5.00 (3.50–5.00)	*U* = 5214.500 ***p* < 0.001**
2–7 days	58	4.71 (3.86–5.00)	
Time from first examination to start of home care			
1 day	242	5.00 (3.50–5.00)	*U* = 5689.500 ***p* < 0.001**
2–7 days	64	4.71 (3.86–5.00)	
Does the home health team call you before they arrive?			
Yes	281	4.92 (3.50–5.00)	*U* = 2254.000 ***p* = 0.001**
No	25	5.00 (4.00–5.00)	

^a^ The Mann–Whitney *U* test was used to compare two independent groups, and the Kruskal–Wallis test was used to compare more than two groups.

^b^ Groups where the difference originates (Bonferroni test).

^c^ *Note:* Bold values indicate statistical significance of *P* < 0.05.

A weak negative correlation was seen between the duration of health care service received following the application and the overall satisfaction of the participants (*p* = 0.001, Table 7). There was no statistically significant relationship observed between the participants' age, monthly income, number of meals per day and satisfaction (*p* > 0.05).

**TABLE 7:** Correlation of participants' home health care satisfaction scores with various variables.

Home health care satisfaction score		
	*r*	*p* *
Age	−0.040	0.481
Monthly income	0.036	0.526
Number of individuals living at home	0.333	**< 0.001**
Duration of receiving health care (years)	0.013	0.826
Number of daily meals	−0.092	0.108
How many days did it take for the home care service to come for the first examination visit after the initial application? (days)	−0.200	**< 0.001**
Time from first examination to start of home health care (days)	−0.193	**0.001**
Caregiver's age	−0.057	0.324

*Note:* Bold values indicate statistical significance of *p* < 0.05.

* Spearman correlation analysis.

## 4 Discussion

The prevalence of home care services is increasing globally, including in Turkey, as a result of the ageing population and the rise in chronic illnesses (Polat et al. 2016). This study analysed the sociodemographic characteristics of patients receiving home care and their caregivers, as well as the level of satisfaction reported by the patients. Additionally, the study explored the factors that affect patient satisfaction.

A significant majority of home care patients' (76.5%) caregivers are female, with 98.0% being relatives or spouses of the dependents. In studies conducted both in Turkey and Korea, more than half of the caregivers are women (Kim and Yeom 2016; Tirgil and Naldoken 2019). It has been reported in the literature that caregivers of home care patients are mostly women (Gozubuyuk 2017; Doganay and Guven 2019). Because home care services, which have roots in history, the burden of family caregiving falls especially on women. This shows that family ties are still very strong in the region.

When the educational status of caregivers is examined, 11.1% are illiterate, 32.0% are literate, 11.8% are primary school graduates, 9.8% are secondary school graduates, 24.8% are high school graduates and 10.5% are university graduates. In Gey and Yarar's study (Gey and Yarar 2019), 18.4% were illiterate, 9.5% were literate, 21.2% were primary school graduates, 11.2% were primary school graduates, 39.1% were high school graduates and 0.6% were university graduates. In Tirgil and Naldoken's study (Tirgil and Naldoken 2019), 5.9% were illiterate, 3.3% were literate, 34.3% were primary school graduates, 16.8% were secondary school graduates, 22.8% were high school graduates and 16.9% were university graduates. In the study conducted in Korea (Kim and Yeom 2016), 15.9% were primary school graduates or below, and 84.1% were secondary school graduates or above. According to Turkish Statistical Institute (TSI) 2022 (Turkish Statistical Institute 2024), the education levels among participants over the age of 15 in Elazig city are as follows: 3.5% are illiterate, 6.2% are literate, 22.3% are primary school graduates, 19.4% are secondary school graduates, 27.3% are high school graduates, 20.2% are university graduates or above. Our study is similar to the literature and the study of TSI. In general, it can be said that the education level of the patient's relatives who care for the patient at home is low.

In this study, participants stated that they learned about home care services most often by getting information from their social circle, and the first thing they applied for home care services was by calling them. In Yesiltas's (Yesiltas 2014), Gey and Yarar's (Gey and Yarar 2019), Tirgil and Naldoken's (Tirgil and Naldoken 2019) studies, they stated that they first received information from the hospital. In Tirgil and Naldoken's study, individuals stated that they most frequently applied by phone (Tirgil and Naldoken 2019). This may be due to the sociocultural differences of the places where the research was conducted.

Almost 90% of the team that conducts the initial examination at participants' homes consists of nurses. In Yesiltas's study, this rate is 97.5% (Yesiltas 2014), in Gey and Yarar's study, this rate is 95.3% (Gey and Yarar 2019). In our study, 79.1% of the participants started receiving service 1 day after the initial first application. In Gey and Yarar's study, 60.9% of individuals started receiving service in 0–3 days (Gey and Yarar 2019). In Tirgil and Naldoken's study, 28.4% started receiving service 1 day after the first application (Tirgil and Naldoken 2019). Differences between studies may be related to the size of the cities where the studies were conducted, and therefore to population and patient density.

In our study, the satisfaction level of participants receiving home care services was found to be quite high (92.8%). Studies conducted in Turkey are also parallel to our study (Guduk et al. 2020). In the study conducted by Gey and Yarar with patients' relatives, when the satisfaction levels of individuals with home care services were examined, it was stated that 65.9% were very good, 26.8% were good and 57.3 were moderately satisfied (Gey and Yarar 2019). In Torun et al.'s study conducted in Ankara, Turkey, the satisfaction rate was 77% (Torun et al. 2016). In the study conducted in Indonesia, 59.5% of individuals were satisfied (Bela et al. 2020). Our research has revealed that participants receiving home care services have a positive attitude towards these services. Determining patient satisfaction is one of the most important indicators of health service quality (Wei et al. 2015). We can conclude that the home care program in Turkey is quite successful in providing needed services to individuals.

In this study, patients' satisfaction did not vary by gender. Studies conducted in Turkey and Sweden were parallel to our study (Guduk et al. 2020; Boström et al. 2022). In another study conducted among palliative care patients, men had higher satisfaction levels (Kamel et al. 2023). In addition, there were studies where home care satisfaction was higher in women (Tirgil and Naldoken 2019).

In this study, there was no difference between age and satisfaction scores. In Tirgil and Naldoken's study, no difference was found between age and satisfaction, similar to our study (Tirgil and Naldoken 2019). There was no relationship between age and satisfaction in a study conducted among palliative care patients and in a study conducted among the elderly in Sweden (Boström et al. 2022; Kamel et al. 2023). In Guduk's study, the satisfaction scores of individuals aged 49 and under were found to be significantly higher (Guduk et al. 2020). The difference between the sample groups may have caused this situation.

Participants' satisfaction did not vary according to marital status. There is no relationship between marital status and satisfaction in both Tirgil and Naldoken's study (Tirgil and Naldoken 2019), and a study conducted among palliative care patients (Kamel et al. 2023). In this study, the satisfaction scores of participants who had children at home and had a large family type were significantly higher. In Torun et al.'s study, satisfaction scores of patients with children were also high (Torun et al. 2016). In our study, the satisfaction of university graduate participants was found to be significantly high. In Tirgil and Naldoken's study (Tirgil and Naldoken 2019) the satisfaction of participants who were secondary school graduates was significantly higher. In a study conducted among palliative care patients, satisfaction levels increased as the education level decreased (Kamel et al. 2023). In a study conducted in Indonesia, it is thought that increasing the level of education will contribute to individuals' better evaluation of the service and increase their satisfaction (Bela et al. 2020).

In this study, the satisfaction of participants with high monthly income levels was significantly higher. On the other hand, in a study conducted in a province in western Turkey, it was found that high income level reduces satisfaction (Torun et al. 2016). Satisfaction scores were higher in those who cared for the patient at home and also worked in another job. In a study examining the satisfaction of caregivers in Iceland, the satisfaction of caregivers who were not employed was higher than those who worked part time (Hjörleifsdóttir et al. 2019). The reason for the difference is; there may be cultural factors, education and health system differences.

The satisfaction scores of the participants who learned about home care services from the social environment and media were significantly higher. In Tirgil and Naldoken's study, the satisfaction of the participants who learned about home care services from someone they knew was higher (Tirgil and Naldoken 2019). Media and social environments influence the satisfaction or dissatisfaction of a people (Alkazemi et al. 2020).

In this study, the satisfaction of the participants who started receiving service within 1 day after the initial application was significantly high. Tirgil and Naldoken's study was also compatible with our study (Tirgil and Naldoken 2019). In this study, no difference was found between the total years received from home care and satisfaction scores. On the other hand, in Tirgil and Naldoken's study, the satisfaction of those who received home care services for more than 4 years was higher (Tirgil and Naldoken 2019). There is a thought that individuals with more experience using home care services possess a deeper level of knowledge on this issue, which is potentially associated with greater satisfaction.

## 5 Conclusion

It is important for healthcare organisations to understand what consumers need or want so that they can meet their care service expectations. It is thought that making home care services more widespread, more qualified and more easily accessible can help both increase patient satisfaction, reduce congestion in hospitals and reduce costs. In this study, it was revealed that almost all of the participants stated that they were satisfied with the use of home care services. Satisfaction with home care services is affected by various factors. It is recommended to use satisfaction surveys periodically to determine the reasons for dissatisfaction among participants who are dissatisfied with the service and to conduct research for improvement. In this way, the quality of home care services can be increased.

There are few studies evaluating home care patients' satisfaction and affecting factors in Turkey. This constitutes the strength of our study. The limitations of the study are that a mixed method was not used and the research was conducted in a single province. More extensive and qualified studies should be conducted in this field.

## Author Contributions

The authors take full responsibility for this article.

## Disclosure

The authors have nothing to report.

## Ethics Statement

Before the research, ethics committee permission was obtained from Firat University Non‐invasive Research Ethics Committee (09.06.2022‐190407) and consent was obtained from the participants.

## Consent

The authors have nothing to report.

## Conflicts of Interest

The authors declare no conflicts of interest.

## Data Availability

Data will be shared with individuals upon request.
